# Evaluations of Biomarker Status Changes between Primary and Recurrent Tumor Tissue Samples in Breast Cancer Patients

**DOI:** 10.1155/2019/7391237

**Published:** 2019-09-10

**Authors:** Thi Hoa Nguyen, Van Hung Nguyen, Thanh Long Nguyen, Cai Qiuyin, Thi Huyen Phung

**Affiliations:** ^1^Department of Medical Oncology 6, Vietnam National Cancer Hospital, Hanoi, Vietnam; ^2^Department of Oncology, Hanoi Medical University, Hanoi, Vietnam; ^3^Division of Epidemiology, Department of Medicine, Vanderbilt Epidemiology Center, Vanderbilt University School of Medicine, Nashville, TN, USA; ^4^Department of Oncology, Vietnam University of Traditional Medicine, Hanoi, Vietnam

## Abstract

**Background:**

Obtaining tumor specimens and re-evaluating targeted markers is recommended, if possible, in breast cancer patients who relapsed after curative treatment. The biomarker status changes in rebiopsied tumors have been demonstrated to have considerable clinical implications.

**Objectives:**

To identify the changes of estrogen receptor (ER), progesterone receptor (PR), and human epidermal growth factor receptor 2 (HER2) status between the primary and recurrent lesions.

**Materials and Methods:**

We conducted a study among 67 patients with recurrent breast cancer, recruited from January 2014 to September 2018 in the Vietnam National Cancer Hospital to compare ER, PR, and HER2 status between the primary and recurrent lesions. For each patient, a specimen of their primary tumor and another specimen of recurrent lesions underwent pathological assessment. Immunohistochemistry (IHC) was performed to determine ER, PR, and HER2 status in both specimens.

**Results:**

Biomarker status conversion rates (in both directions) between primary and recurrent tumors were 26.9% for ER, 38.8% for PR, and 22.4% for HER2. Overall, IHC subtypes (hormone receptor positive, HER2 amplified, and triple-negative) changed in 25 out of 67 (37.3%) cases. Conversion rates were not statistically significantly different between patients with different recurrent sites and times of recurrence. Eight out of 13 initially triple-negative patients (61.5%) had a change to positive status of either ER, PR, or HER2.

**Conclusion:**

A substantial discordance in ER, PR, and HER2 status were observed between primary breast cancer tissues and recurrent lesions. Rebiopsy could bring new therapeutic opportunities in the management of patients with recurrent breast cancer.

## 1. Introduction

Breast cancer has been recognized as the most common type of cancer and the leading cause of malignancy-related mortality in women worldwide [1]. In Vietnam, breast cancer incidence had an age-standardized rate of 29.9 per 100,000 women in 2010, which had doubled over the last two decades [2]. Despite the increasing trend in breast cancer incidence, there have been certain improvements in the prognosis and treatment thanks to the advances in the understanding of related biomarkers and the development of corresponding therapeutic approach [3, 4].

Among various biomarkers, estrogen receptor (ER), progesterone receptor (PR), and human epidermal growth factor receptor 2 (HER2) play important roles in management and prognosis of patients with breast cancer [3]. Approximately 60%–70% of breast cancer patients are hormone-receptor positive and 20%–25% have amplified HER2 [5, 6]. According to the 2013 St Gallen Consensus Conference, based on ER, PR, HER2, and Ki67 status, breast cancer patients are divided into different subtypes, including Luminal A, Luminal B, HER2-amplified, and triple-negative [7]. Patients with different subtypes are treated differently in both early and advanced stages, and have different survival time [3, 8]. For example, in a study on 196,094 breast cancer patients, Luminal A group had a 4 year survival rate of 92.5%, followed by Luminal B (90.3%), HER2-amplified (82.7%), and finally worst survival for triple-negative subtype (77.0%) [9]. Patients with positive hormone receptor could be treated with endocrine therapy and those with HER2 overexpression have survival benefits from trastuzumab and/or pertuzumab treatment [10]. Meanwhile, in the triple-negative group, treatment options are usually limited to chemotherapy, and the polyadenosine diphosphate-ribose polymerase (PARP) inhibitor olaparib for selected cases with BRCA mutation [11]. Recently, androgen receptor (AR) expression has been evaluated in breast cancer, in which AR is associated with cell proliferation and metastasis in ER-negative breast cancer [12]. This evidence supports AR-targeted therapies, including bicalutamide and enzalutamide might be useful in patients with triple-negative breast cancer [12]. Therefore, biomarker status assessment has a significant clinical utility in guiding treatment decision-making in not only newly diagnosed breast cancer but also in recurrent settings. Recently, some studies have demonstrated a remarkable rate of conversion of these biomarker status in patients with recurrent breast cancer after curative treatment [13–17]. However, the conversion rate of each biomarker is inconsistent in the previous studies. The changes in receptor status, meanwhile, can result in changing the treatment approach, and also is a clinically significant prognostic factor of overall survival [15, 18, 19]. Therefore, international guidelines have encouraged to perform biopsy of recurrent lesions to re-evaluate biomarker status [10].

However, in Vietnam, rebiopsy of recurrent lesions has not been routinely performed. The objective of this study is to evaluate the changes of ER, PR, and HER2 status between the primary and recurrent lesions in Vietnamese patients with breast cancer.

## 2. Materials and Methods

### 2.1. Study Design

This is a retrospective and observational study, conducted at the Vietnam National Cancer Hospital. Convenience sampling method was used to enroll patients during a 4 year period from January 2014 to September 2018. This study was approved by the research committee of the Vietnam National Cancer Institute.

### 2.2. Study Population

We included patients who were diagnosed with recurrent breast cancer after curative treatment at our institution during the study period. The staging of primary tumor was based on the AJCC Cancer Staging Manual, 7^th^ edition [20]. All patients were clinically or radiologically suspected of recurrence by their primary oncologists and underwent biopsy or surgical resection of the recurrent lesions for confirmation. Recurrent lesions (RL) were defined as any local, regional, or distant recurrence. For superficial lesions, core or excisional biopsy was performed. For internal lesions, the most amenable site of biopsy was determined in consultation with an interventional radiologist, and core biopsy was carried out under radiologic guidance (i.e., ultrasound for liver lesions and axillary or supraclavicular lymph nodes, CT scan for lung lesions, mediastinal lymph nodes, and bone lesions). One patient with seizure and suspected brain tumor in MRI scan underwent surgery for brain tumor resection which revealed a metastasis originating from the breast. Tissue samples were evaluated by hematoxylin and eosin staining and an immunohistochemical panel that includes ER, PR, HER2, and Ki67. Exclusion criteria included (1) patients with de novo metastatic breast cancer, (2) patients with contralateral tumor or pathological results suggesting a new primary tumor, and (3) patients with incomplete pathological and immunohistochemical information.

### 2.3. ER, PR, and HER2 Determination

**Figure 1:**
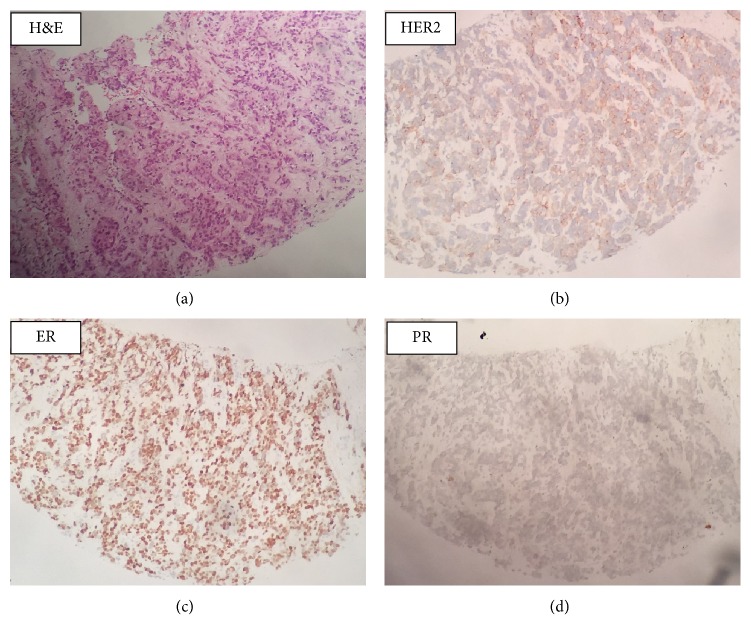
Representative examples of IHC analysis of the studied biomarkers on a biopsied specimen. ER was markedly expressed (ER score 2+) and PR staining status was negative. The staining score of HER2 was 2+ (FISH test showed that the tumor was HER2-amplified). H&E, hematoxylin and eosin stain; IHC, immunohistochemistry; ER, estrogen receptor; PR, progesterone receptor; HER2, human epidermal growth factor receptor 2; FISH, Fluorescence in situ hybridization.

The ER, PR, and HER2 status for the primary tumors were obtained from medical chart/pathology report reviews. The rebiopsy tumor tissue samples from recurrent lesions were stained for ER, PR, and HER2 using formalin-fixed and paraffin-embedded (FFPE) sections following Avidin-Biotin Complex (ABC) method, in which tetravalent strept (avidin) and biotinylated antibodies were used. ER, PR, and HER2 classifications were made based on the American Society of Clinical Oncology/College of American Pathologists guideline recommendations for IHC testing of ER, PR, and HER2 in breast cancer [21, 22]. If ≥1% of tumor cells show positive ER/PR staining of any intensity, the ER/PR interpretation is positive. Negative hormone receptor is defined as <1% of tumor cells with ER/PR staining of any intensity. Meanwhile, a standard 0–3+ scoring system was used to evaluate HER2 status, in which 0 and 1+ scores were considered negative and 3+ was considered positive. If IHC HER2 scored 2+, HER2 FISH test was conducted. HER2/CEP17 ratio ≥2 was defined as HER2 amplified (Figure 1).

### 2.4. Subtype Definition

The patients were divided into three IHC subtypes based on primary tumor, including HR-positive (ER positive and/or PR positive and HER2 negative), HER2-amplified (HER2 positive/any ER, PR status), and triple-negative (ER negative, PR negative, and HER2 negative).

### 2.5. Statistical Analysis

All data were presented descriptively as a median (interquartile range) or number (percentage). Comparisons between different groups were done using Fisher's exact test or Chi square test where appropriate. *p* values < 0.05 were considered statistically significant. Data were analyzed using STATA SE 12.0 for Windows (STATA Corp., College Station, TX 77845).

## 3. Results

### 3.1. Patients' Baseline Characteristics

During the study period, a total of 67 patients were diagnosed with recurrent breast cancer at our institution. The IHC profile of primary tumors was ER/PR-positive in 30 (44.8%) patients, HER2-amplified in 24 (35.8%) patients, and triple-negative in 13 (19.4%) patients.

A summary of patient characteristics is presented in Table 1. Ductal carcinoma accounted for a majority of cases (80.6%, 54/67), followed by lobular carcinoma (9.0%, 6/67). After initial diagnosis, all patients underwent surgery, and 61/67 (91.0%) patients then received either adjuvant chemotherapy, endocrine therapy, or target therapy prior to recurrences. The median time from diagnosis of the primary tumor to identification of recurrences was 45 months (interquartile range (IQR) 24–59 months). The most common site of recurrence was regional lymph nodes (36/67, 53.7%), followed by chest wall (17/67, 25.4%).

### 3.2. Change in Receptor Profile between the Primary and Recurrent Lesions

The concordance and discordance of tumor biomarkers between primary and recurrent lesions are shown in Tables 2 and 3. Among the three receptors, PR status had the highest conversion rate (38.8%, 95% CI 26.8%–50.8%) between primary and recurrent lesions; the majority of which was from PR-positive to PR-negative 17/67 (25.4%), compared to 9/67 (13.4%) in the opposite direction. ER status conversion was seen in 26.9% patients (95% CI: 16.0%–37.8%); however, it comprised comparable numbers of gain vs. loss (8 vs. 10 patients, respectively). The overall conversion rate for HR was 25.4% (17/67 patients, 95% CI: 15.5%–37.5%), similarly distributed between two directions (8 gains vs. 9 losses). Among the discordant subset, ER and PR conversions were most commonly observed in the HR-positive group (38.9%, 7/18 for ER and 69.2%, 18/26 for PR) (Table 3). There were 5/13 (38.5%) triple-negative patients that switched hormone receptor profile (either ER or PR).

In terms of HER2, the conversion rate was 22.4% (95% CI: 12.1%–32.6%), including 5 (7.5%) patients from positive to negative and 10 (14.9%) patients in the reverse. HER2 gain proportions were 20% (6/30) and 30.8% (4/13) in HR-positive and triple-negative groups, respectively.

With the above conversion of receptor status, the IHC subtype was changed in 25/67 (37.3%) patients. Changing from HR-positive to HER2 amplified and to Triple-negative had the highest frequency (6/67 patients in each change, 9.0%). Meanwhile, changes from HER2 amplified to HR-positive, from triple-negative to HR-positive and to HER2 amplified occurred in 4 patients (6.0%) each. Only 1 patient (1.5%) switched from HER2 amplified to triple-negative. In contrast, a change to positive of either ER, PR, or HER2 was observed in 8/13 triple-negative patients (61.5%).

Regarding the biopsy of recurrent lesions, changes in ER, PR, and HER2 status of locoregional recurrences were recorded in 26.7%, 40.0%, and 20.0%, respectively. Among distant metastasis biopsies, discordance rate was also highest in PR status (36.4%), followed by ER and HER2 (27.3% each) (Table 4).

### 3.3. Effects of the Duration between Primary and Recurrent Disease and the Recurrent Site on Conversion Rates

The proportions of receptor discordance were similar between locoregional and distant metastatic sites, which was presented in all three IHC subtype groups (*p*-values > 0.05, see details in Table 4). Significant differences were also not observed between patients with recurrences that occurred >36 months and ≤36 months after primary disease (ER: 32% vs. 27%; PR: 32 vs. 43%; HER2: 20 vs. 24%, *p*-values > 0.05).

## 4. Discussion

Our study included 67 cases of recurrent breast cancer after curative treatment. RL biopsies with IHC showed considerable rates of receptor status conversions, including 26.9% in ER, 38.8% in PR, and 22.4% in HER2. There were no statistical differences in conversion rates in regard to different sites and times of recurrence. Eight out of 13 triple-negative patients (61.5%) had a change to positive of either ER, PR, or HER2 status compared to the primary tumors. To our knowledge, this is one of the few reports in Vietnam as well as in other developing countries [18, 23–25], focusing on the conversion of breast cancer biomarkers between primary tumors and recurrence lesions.

Biopsy of metastatic and recurrent lesions in breast cancer plays an important role, not only to achieve a final diagnosis, but also to re-evaluate ER, PR, and HER2 status. Evidence has shown that the conversion of these markers between primary and RL could be useful in the clinical management of patients with breast cancer [16, 17]. Previous studies reported that 39%–46% of treatment plans were modified according to the conversion of rebiopsy IHC [16, 17]. In the Breast Recurrence In Tissues Study (BRITS) which prospectively investigated the receptor status of 137 paired tissue samples of primary and recurrent tumors, ER, PR, and HER2 status were changed in 10%, 25%, and 3% patients, respectively [16]. Meanwhile, a prospective cohort study (DESTINY) of 121 women with recurrent or metastatic breast cancer reported a conversion rate of 16% in ER, 40% in PR, and 10% in HER2 [17]. A pooled analysis of these two studies yielded ER, PR, and HER2 discordant rates of 13%, 31%, and 6%, respectively [15]. These proportions are lower than results from our study (26.9% for ER, 38.8% for PR, and 22.4% for HER2). The variation of discordance rates between different studies might be due to laboratory artifacts, tissue handling and processing, time of specimen preparation as well as result interpretation [13, 15]. In a prospective study on 184 patients with recurrent or metastatic breast cancer in Spain, receptor discordance rates were different when tested at central vs local laboratories (13% vs. 21% for ER, 28% vs. 35% for PR, and 3% vs. 16% for HER2) [13]. In a prospective observational study in 178 patients, the discordance rates between primary and metastasis lesions were 13%, 28%, and 3% for ER, PR, and HER2, respectively. In our study, rates of ER and HER2 status conversion from negative to positive were both approximately 15%, which are slightly higher than in other studies [15, 26]. In these patients, re-evaluation of tumor biomarkers had provided new treatment options, especially for triple-negative group. Moreover, according to Vietnam Law in Health Insurance, 80% of the treatment cost can be covered by insurance in most treatment modalities, but for certain drugs, patients need to pay at least one-half of the cost, such as trastuzumab (US$10,000–40,000 per individual) [2]. If there is conversion to HER2-negative in recurrent disease, continuation of trastuzumab might not be as effective as expected and cause major economic burden on patients, especially those living on poverty. Therefore, rebiopsy of recurrent lesions might be necessary to assess tumor receptor status.

There are several possible mechanisms for the conversion in ER, PR, and HER-2 expression. Firstly, technical artifacts and the variability in the accuracy of IHC tests may contribute to the difference of biomarker status between primary and recurrent tumors [27]. However, if laboratory issues were the main cause of status changes, the discordance rates would be expected to be approximately equal among ER, PR, and HER2 as well as between two directions. Meanwhile, in our study, the conversion rate was significantly different among ER, PR, and HER2 (26.9%, 38.8%, and 22.4%, respectively), which is consistent with previous studies [15–17]. Another possible etiology of receptor status changes is the clonal genome evolution and biological heterogeneity of the tumor, in which the more aggressive cell clones might be more likely to be involved in the micro-metastatic and recurrent process [28–30]. Besides, the discordance might also be a result of a biological drift due to clonal selection under the pressure of therapy. Some studies demonstrated the association between the use of hormonal therapy and the disappearance of ER/PR-positive cells [31, 32] as well as the effect of previous trastuzumab treatment on HER2 conversion [33, 34]. Finally, although newly acquired genomic mutations appear to be rare, it is still possible that the genuine changes in tumor biology can contribute to receptor status discordance [35].

Discordances of receptor status between primary tumor and recurrent lesions may lead to difficulty in determining tumor status and planning treatment accordingly [15]. In this case, reanalyzing of the primary specimen would be helpful to confirm the tumor profile. In the DESTINY study, three primary tumors initially categorized as ER-negative were found to be ER-positive after reanalysis [17]. Two triple-negative tumors in the initial pathology reports were eventually ER-positive after reassessment [17]. This suggests the clinical importance of storing primary tumor specimens to recheck the receptor profile of the tumor when needed, which has not been well-recognized in resource-limited settings, like in Vietnam.

Our results, consistent with previous studies, did not observe any associations between receptor conversion and the duration from primary tumor diagnosis to recurrence, sites of recurrence, or tumor molecular subtypes [13, 15]. This, again, emphasizes the importance of rebiopsy of recurrent lesions in patients with breast cancer, regardless of the above-mentioned characteristics. Our study has some limitations. Firstly, due to the retrospective design and inadequate storage capacity on initial specimens, we were unable to reanalyze to confirm primary tumor receptor status. Secondly, effects of receptor conversion on overall survival and treatment alteration were not assessed in this study due to limited follow-up duration. Finally, the sample size was not large enough to evaluate the effect of previous therapeutic approach on tumor biomarker conversion. Therefore, there is a need for further prospective and more comprehensive studies to thoroughly investigate the receptor conversion rate and its impacts.

## 5. Conclusion

There is a substantial conversion rate of receptor status between primary and recurrent tumors in breast cancer. Rebiopsy could help confirm the recurrence and may play an important role in clinical management. The conversion from receptor-negative to positive may provide new therapeutic opportunities for patients with recurrent breast cancer, including endocrine and targeted therapies for those who were previously not indicated.

## Figures and Tables

**Table 1:** Patient demographics and clinical characteristics.

Characteristics	Number (*N* = 67)	Percentage
*At time of initial diagnosis*		
Age (median (IQR))	44	(38–53)
Menopause status		
Pre-menopause	38	56.7%
Post-menopause	29	43.3%
Stage at diagnosis		
0	2	3.0%
I	11	16.4%
II	27	40.3%
III	27	30.3%
Pathological types		
Ductal	54	80.6%
Lobular	6	9.0%
Other	7	10.4%
Therapy		
Chemotherapy	53	79.1%
Endocrine therapy	37	55.2%
Trastuzumab	5	7.5%
*At time of recurrence*		
Duration from primary to recurrent disease		
<24 months	17	25.4%
24–36 months	8	11.9%
>36–60 months	28	41.8%
>60 months	14	20.9%
Locoregional recurrence	48	71.6%
Chest wall	17	25.4%
Regional lymph nodes	36	53.7%
Distant metastasis		
Lung	15	22.4%
Liver	5	7.5%
Bone	10	14.9%
Mediastinal lymph node	9	13.4%
Data: distant metastasis/other	5	7.5%
Site of biopsy		
Locoregional	45	67.2%
Lung	8	11.9%
Liver	4	6.0%
Bone	2	3.0%
Brain	1	1.5%
Mediastinal lymph node	1	1.5%
Others	6	9.0%

**Table 2:** Distribution of ER, PR, and HER2 status between the primary tumor and recurrent lesions (*N* = 67).

Biomarkers	Concordance*N* (%)		Discordance*N* (%)		Total discordance*N* (%)
	PL (+)	PL (−)	PL (+)	PL (−)	
	RL (+)	RL (−)	RL (−)	RL (+)	
ER	27 (40.3%)	22 (32.8%)	8 (11.9%)	10 (14.9%)	18 (26.9%)
PR	11 (16.4%)	30 (44.8%)	17 (25.4%)	9 (13.4%)	26 (38.8%)
HER2	19 (29.7%)	33 (49.3%)	5 (7.5%)	10 (14.9%)	15 (22.4%)

PL: Primary lesions; RL: Recurrent lesions.

**Table 3:** Conversion rates of receptor status between primary tumor and metastasis.

Markers	Total	HR-positive *N* (%)	HER2 amplified *N* (%)	Triple-negative *N* (%)
*ER*				
No conversion	49	23 (76.7%)	18 (75.0%)	8 (61.5%)
From (+) to (−)	8	6 (20.0%)	2 (8.3%)	0
From (−) to (+)	10	1 (3.3%)	4 (16.7%)	5 (38.5%)
Total	67	30 (100%)	24 (100%)	13 (100%)
*PR*				
No conversion	41	12 (40.0%)	20 (83.3%)	9 (69.2%)
From (+) to (−)	17	14 (46.7%)	3 (12.5%)	0
From (−) to (+)	9	4 (13.3%)	1 (4.2%)	4 (30.8%)
Total	67	30 (100%)	24 (100%)	13 (100%)
*HER2*				
No conversion	52	24 (80.0%)	19 (79.2%)	9 (69.2%)
From (+) to (−)	5	0	5 (20.8%)	0
From (−) to (+)	10	6 (20.0%)	0	4 (30.8%)
Total	67	30 (100%)	24 (100%)	13 (100%)

**Table 4:** Distribution of ER, PR, and HER2 status according to biopsied site.

Sites of biopsy	Total	Change in ER		Change in PR		Change in HER2 status	
		Discordant*N* (%)	Concordant*N* (%)	Discordant*N* (%)	Concordant*N* (%)	Discordant*N* (%)	Concordant*N* (%)
Locoregional lesions	45	12 (26.7%)	33 (73.3%)	18 (40.0%)	27 (60.0%)	9 (20.0%)	36 (80.0%)
Metastasis lesions	22	6 (27.3%)	16 (72.7%)	8 (36.4%)	14 (63.6%)	6 (27.3%)	16 (72.7%)
Lung	8	3 (37.5%)	5 (62.5%)	2 (25.0%)	6 (75.0%)	2 (25.0%)	6 (75.0%)
Liver	4	0	4 (100%)	0	4 (100%)	1 (25.0%)	3 (75.0%)
Bone	2	1 (50%)	1 (50%)	1 (50%)	1 (50%)	0	2 (100%)
MLN	1	0	1 (100%)	1 (100%)	0	1 (100%)	0
Others	7	2 (28.6%)	5 (71.4%)	4 (57.1%)	3 (42.9%)	2 (28.6%)	5 (71.4%)

MLN: Mediastinal lymph nodes.

## Data Availability

The datasets used and/or analyzed during the current study available from the corresponding author on reasonable request.
